# P2X-Receptor Antagonists Inhibit the Interaction of *S. aureus* Hemolysin A with Membranes

**DOI:** 10.3390/toxins9100332

**Published:** 2017-10-19

**Authors:** Markus Schwiering, Matthias Husmann, Nadja Hellmann

**Affiliations:** 1 Institute for Molecular Biophysics, Jakob-Welder-Weg 26, University of Mainz, 55128 Mainz, Germany; schwiering@uni-mainz.de; 2 Institute of Medical Microbiology and Hygiene, University Medical Center, Johannes Gutenberg-University Mainz, Hochhaus am Augustusplatz, 55131 Mainz, Germany; husmann@uni-mainz.de

**Keywords:** hemolysin, pore forming toxin, *Staphylococcus aureus*, P2XR, P2XR-antagonists, erythrocytes, HaCaT-cells, liposomes, oligomerisation

## Abstract

The pore forming hemolysin A, Hla, is a major virulence factor of *Staphylococcus aureus*. Apparently, 1–2 pore(s) per cell suffice(s) to cause cell death. Accumulated experimental evidence points towards a major role of ATP-gated purinergic receptors (P2XR) for hemolysis caused by Hla, complement and other pore forming proteins, presumably by increasing membrane permeability. Indeed, in experiments employing rabbit erythrocytes, inhibitory concentrations of frequently employed P2XR-antagonists were in a similar range as previously reported for erythrocytes of other species and other toxins. However, Hla-dependent hemolysis was not enhanced by extracellular ATP, and oxidized adenosinetriphosphate (oxATP) had only a minor inhibitory effect. Unexpectedly, P2XR-inhibitors also prevented Hla-induced lysis of pure lipid membranes, demonstrating that the inhibition did not even depend on the presence of P2XR. Fluorescence microscopy and gel-electrophoresis clearly revealed that P2XR-inhibitors interfere with binding and subsequent oligomerisation of Hla with membranes. Similar results were obtained employing HaCaT-cells. Furthermore, calorimetric data and hemolysis experiments with Hla pre-treated with pyridoxal phosphate-6-azophenyl-2′,4′-disulfonic acid (PPADS) showed that this compound directly binds to Hla. Our results call for a critical re-assessment of the appealing concept, which suggests that P2XR are general amplifiers of damage by pore-forming proteins.

## 1. Introduction

Pathogenic bacteria employ pore forming toxins (PFTs) to subvert, invade, or destroy host target cells. The factors defining the toxin-susceptibility of different cell types and the species variation for a given cell-type are only partially understood. This limits the development of anti-infective strategies targeting PFTs, which are increasingly in demand due to a dwindling pool of effective antibiotics. Generally, the susceptibility of host cells to a given toxin seems to depend on the presence of specific high-affinity receptors for binding of toxin monomers, subsequent oligomerisation, and pore formation [1,2]. Further, the efficiency of the host cell’s repair mechanisms co-determines the outcome of an attack [3]. Yet another mechanism that may play a role for the impact of pore-forming toxins on target cells is the enhancement of damage by purinergic signaling [4]. This mechanism is supported by several lines of evidence: in case of RTX-family-toxins (Repeats in ToXin-family), hemolysis was inhibited by inhibitors specific for ATP-gated purinergic receptors (P2XRs) or upon depletion of extracellular ATP [5,6]. Since P2XRs are activated by ATP, these observations point towards a role of these receptors for enhancing the effect of RTX-toxin-induced hemolysis. Increased levels of extracellular ATP were measured upon treatment of erythrocytes with the RTX-toxins HlyA (hemolysin A from *E. coli*), and LTX (Leukotoxin from *Aggregatibacter Actinomycetemcomitans*), and based on the absence of effect of various inhibitors, it was concluded that this increase could not be accounted for by egress through cellular membrane channels, but was likely due to leakage through toxin pores [7]. Furthermore, leakage of ATP from liposomes loaded with ATP was observed upon addition of RTX toxins, apparently supporting the idea that ATP (mass 507.18 g/mol) can pass through the RTX toxin pores. Notably, under the same conditions, egress of calcein (mass 622.55 g/mol) was not detectable. The concept that P2XR-signaling enhances the hemolytic effect of pore formers was extended to other proteins, including Hla from *S. aureus* [8] and complement [9]. The collective data were interpreted to indicate that P2XR-signalling is a general enhancer mechanism in hemolysis.

These reports led us to investigate whether P2XR-signaling is also relevant for Hla-dependent toxicity towards spontaneously transformed human adult skin keratinocytes (HaCaT). Our initial finding that pyridoxal phosphate-6-azophenyl-2′,4′-disulfonic acid (PPADS) inhibited Hla-dependent cytotoxicity seemed to support this idea. However, subsequent binding assays indicated that PPADS already inhibited the interaction of Hla with cells. This raised the question of whether Hla-dependent action on erythrocytes, detected as hemolysis, might be affected by P2XR-inhibitors through similar non-canonical mechanisms. Accordingly, we decided to re-investigate the role of P2XR-signalling for Hla-dependent lysis of erythrocytes. We chose rabbit erythrocytes and Hla from *S. aureus* as a model. Rabbit erythrocytes are the most sensitive erythrocytes with respect to Hla-induced hemolysis. Early experiments indicated that the amount of Hla molecules irreversibly bound to rabbit erythrocytes are about 10 monomers per cell at a level of 50% lysis (after 6 h), corresponding to 1–2 pores [10]. It seems plausible that in order to obtain hemolysis by such a small number of pores, cellular mechanisms enhancing the permeability of the plasma-membrane could be involved. Just as in the studies mentioned above, we investigated the extent of hemolysis in the absence or presence of inhibitors and activators of P2XRs. Furthermore, in order to exclude unspecific interactions between the P2XR-inhibitors, lipid-membranes and Hla, we also studied calcein efflux from liposomes in the presence of these substances. In addition, oligomerisation of Hla in the presence of inhibitors was investigated by gel-electrophoresis, using liposomes or erythrocyte membranes, supplemented by a calorimetric study of the PPADS/Hla binding in absence of liposomes or cells. The results of this study indicate that P2XR-antagonists interfere with binding and/or oligomerisation of Hla to target membranes, raising doubts that P2XRs play a general role in pore-forming toxin-dependent hemolysis.

## 2. Results

### 2.1. PPADS Reduces Cytotoxicity of Hla for HaCaT-Cells and Binding of the Toxin

**Figure 1:**
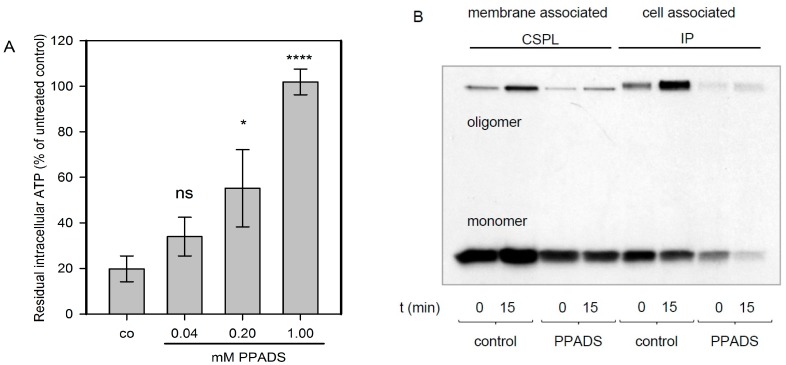
Pyridoxal phosphate-6-azophenyl-2′,4′-disulfonic acid (PPADS) protects HaCaT-cells from Hla-dependent loss of ATP and inhibits Hla oligomerisation. Panel (**A**): Human adult skin keratinocytes (HaCaT-cells) were treated with 6 nM Hla for 2 h in the presence or absence of PPADS (pyridoxal phosphate-6-azo(benzene-2,4-disulfonic acid) at the indicated concentrations. Subsequently cellular ATP was measured. Shown are mean ± standard deviation of *n* = 3 independent assays. Differences between control samples (co; i.e., HaCaT cells with Hla only) and samples receiving additionally 1 mM or 200 µM PPADS are significant as assessed by ANOVA multiple comparison and Tukey’s post-test: ns (not significant) denotes *p* > 0.05; * denotes *p* ≤ 0.05 and **** denotes *p* ≤ 0.0001; Panel (**B**): HaCaT-cells were incubated in absence (control) or presence of 1 mM PPADS for 30 min at 37 °C, followed by incubation for 40 min on ice with radioactive Hla (about 30 nM). Directly after washing (0 min) or after a subsequent incubation at 37 °C for 15 min, bound Hla was determined. Cell-associated Hla was immune-precipitated from the pellet (IP), while membrane-associated Hla was precipitated employing surface-biotinylation followed by application on a streptavidin-column (CSPL). Fluorographic analysis of the SDS-gel separated bands show the presence of two bands, the monomeric Hla at about 33 kDa, and the oligomeric form between 200 and 250 kDa. The experiment was repeated with virtually identical results. The reduced intensity of the bands in presence of PPADS indicate a reduced level of cell-associated and membrane-associated monomers and oligomers.

In order to elucidate the role of P2XRs for nucleated cells, HaCaT-cells that had been used in several previous studies with Hla [11] were used. In the case of nucleated cells, an early cytotoxic effect that has been consistently observed with all of the membrane pore-forming agents investigated is a drop of cellular ATP-levels, which is thought to result from mitochondrial failure as a consequence of dissipating ion gradients. If P2XRs were relevant for Hla-dependent cytotoxicity, PPADS, a potent P2XR-inhibitor, should prevent this drop of ATP. We observed that HaCaT-cells, exposed for 2 h to Hla (6 nM), lost about 80% of their cellular ATP, but in the presence of 1 mM PPADS, this effect was completely blocked; about 40% inhibition was achieved with 200 µM of the inhibitor (Figure 1A). This finding was reminiscent of a recent observation by Nagahama et al., who observed for human leukemia monocytic cells (THP1-cells), that PPADS inhibited the cytotoxicity of *C. perfringens* beta-toxin, a small PFT related to Hla [12].

In order to elucidate the mechanism of PPADS-mediated protection from Hla-dependent cytotoxicity towards HaCaT-cells, we investigated whether PPADS affects the interaction of Hla with the target cell membrane. To this end, we studied the binding of ^35^S-Hla to HaCaT-cells. Gel-electrophoresis of whole cell lysates revealed that the total amount of toxin and the level of oligomer formation on these cells is markedly reduced in the presence of 1 mM PPADS (Figure 1B). The amount of membrane-associated monomers and SDS-stable oligomers was reduced by PPADS in presence of the inhibitor, whether probed directly after Hla incubation on ice and washing (0 min), or after 15 min of further incubation at 37 °C. The same was observed when comparing the total cell associated Hla amount, which also includes the fraction located in the cytosol. These data indicate that PPADS reduces Hla-binding and/or oligomerisation on HaCaT-cells, which will result in a lower number of pores and reduced cytotoxicity. Since this raises the question whether similar mechanisms may apply to erythrocytes and since the concept of P2XR-enhanced lysis has been put forward based on experiments with these cells, we extended our study accordingly.

### 2.2. Hla-Dependent Lysis of Rabbit Erythrocytes is only Partially Altered by Modulation of *P2XR* Function

**Figure 2:**
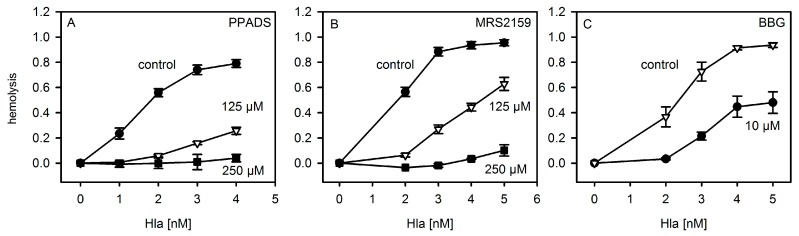
P2XR-inhibitors inhibit Hla-dependent hemolysis of rabbit erythrocytes. Rabbit erythrocytes were incubated for 60 min at 37 °C with three different inhibitors of ATP-gated purinergic receptors (P2XRs): Panel (**A**): PPADS (pyridoxal phosphate-6-azo(benzene-2,4-disulfonic acid), number of experiments: control: *n* = 5; 125 µM: *n* = 3, 250 µM: *n* = 2); Panel (**B**): MRS2159 (a PPADS derived inhibitor) (control: *n* = 4; 125 and 250 µM: *n* = 2 each; Panel (**C**): BBG (Brilliant Blue G) (inhibitor and control: *n* = 3 each). Controls denote the hemolysis curve in absence of inhibitor.

First, we repeated the experiments with P2X-receptor antagonists on hemolysis following the protocols published for murine and horse erythrocytes [8]. As shown in Figure 2, the general P2XR-antagonist PPADS and the analogue MRS2159, which inhibits P2XR1 with a higher efficiency than P2XR7, inhibited Hla-induced hemolysis of rabbit erythrocytes, reminiscent of previous results with mouse erythrocytes. Notably, the inhibition of hemolysis by antagonists was most efficient at moderate Hla concentrations, which is again in line with previous results. The P2XR7-specific antagonist BBG (Brilliant Blue G) also reduced Hla-dependent hemolysis, albeit somewhat higher concentrations of the inhibitor were required as compared to a previous report [8].

**Figure 3:**
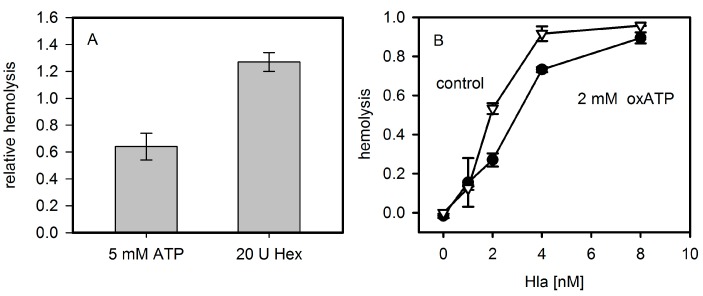
Effect of ATP and analogues on hemolysis. Panel (**A**): Rabbit erythrocytes were mixed with 4 nM Hla with or without extracellular ATP and hemolysis was measured after 15 min at 37 °C. The importance of extracellular bulk ATP was additionally investigated by incubation of erythrocytes in absence and presence of 20 units of hexokinase for 60 min at 37 °C. Lysis levels were normalized to the value in absence of ATP or hexokinase, respectively; Panel (**B**): Rabbit erythrocytes were incubated for 60 min at 37 °C in presence of 2 mM of oxidized ATP (oxATP) and various concentrations of Hla. Number of experiments: ATP and hexokinase: triplicates; oxATP (control: *n* = 2; oxATP: *n* = 4).

The addition of extracellular ATP decreased Hla dependent hemolysis of rabbit erythrocytes after 30 min of incubation (Figure 3A). This is in line with the previous observation that exogenous ATP leads to a significant decrease of Hla-dependent hemolysis at times >15 min in mouse erythrocytes [8]. However, at very early points in time (2.5 min) it had been shown for Hla-induced lysis of mouse erythrocytes that upon addition of 3 mM ATP, an increase from 4% hemolysis to 6% occurs. We could not observe a significant ATP-dependent enhancement with rabbit erythrocytes and Hla at comparable conditions. Similarly, removing extracellular ATP by hexokinase or apyrase was reported to have a strong inhibitory effect on the hemolysis caused by LTX or HlyA [5,6]; hexokinase was also reported to inhibit the effect of Hla in the case of mouse erythrocytes [8]. In contrast, lysis of rabbit erythrocytes by Hla was not significantly affected by hexokinase; the addition of this ATP-consuming enzyme, rather, increases hemolysis slightly (Figure 3A). Along these lines, the competitive P2XR7-specific inhibitor oxATP, even at a concentration of 2 mM, had a considerably less drastic effect on rabbit erythrocytes (Figure 3B) than reported for mouse erythrocytes [8].

### 2.3. P2X-Receptor-Antagonists Inhibit Binding to Erythrocytes and Formation of Oligomers

**Figure 4:**
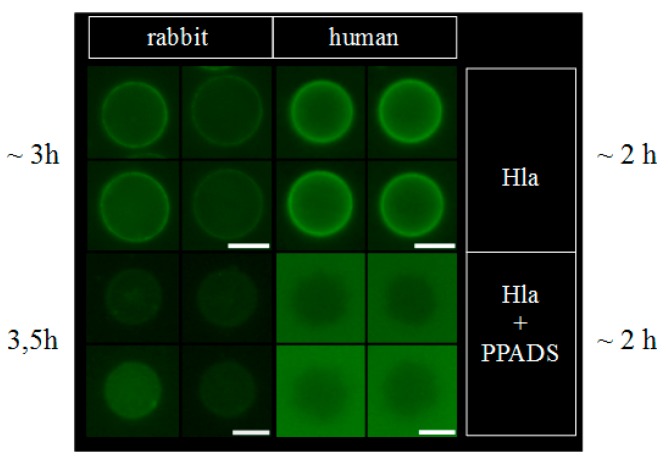
PPADS reduces binding of Hla to erythrocytes. Erythrocytes were treated with 2.5 µM fluorescently labeled Hla in presence or absence of 500 µM PPADS. After 2–3 h of incubation, cell-bound Hla was observed under the microscope. Under these conditions, all of the erythrocytes are lysed. The level of binding can be estimated by comparison of the background fluorescence with the fluorescence of cell-bound Hla. Scale bars indicate 5 µm.

**Figure 5:**
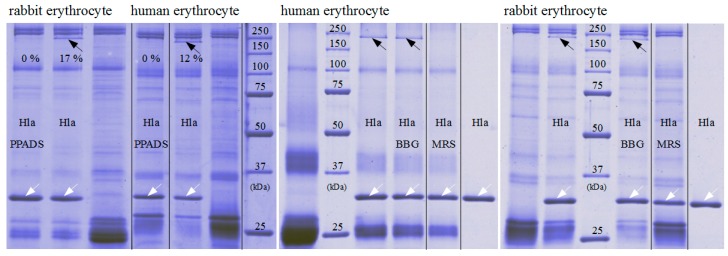
Impact of P2XR-antagonists on Hla-oligomerisation on erythrocytes. Hla-oligomer formation on erythrocyte membranes is strongly reduced in presence of 500 µM PPADS or MRS2159, but not in presence of 20 µM BBG. The white arrows indicate the position of Hla-monomers, the black arrows SDS-stable Hla-oligomers; last lanes in the right two gels are controls with pure Hla without binding partner. The Hla concentration is 2.5 µM in each sample. The given percentage indicate the oligomeric species relative to the total amount.

That hexokinase and ATP did not have the expected effect on Hla-dependent lysis of rabbit erythrocytes, while P2X-receptor antagonists did, and the observation that less Hla is bound to HaCaT-cells in presence of PPADS, fueled the idea that a non-canonical effect of P2X-receptor antagonists was involved: possibly direct interference with the interaction of Hla with the erythrocyte membrane causes the reduction in hemolysis. Accordingly, we studied binding of Hla to rabbit erythrocytes in the presence of P2X-receptor antagonists. In a first approach, we analyzed the effect of PPADS on the overall binding of fluorescently labeled Hla to erythrocytes by fluorescence microscopy. As shown in Figure 4, the fluorescence intensity of the erythrocytes is reduced if PPADS was present. Thus, this indicates that in the presence of PPADS, less Hla is located on the erythrocytes. The effect is very clear in case of human erythrocytes, while in the case of rabbit erythrocytes it is rather subtle. Secondly, we incubated rabbit erythrocytes with Hla in the presence or absence of antagonists and analyzed the formation of sodium dodecyl sulfate (SDS)-stable oligomers by SDS-PAGE. Although Hla-oligomer formation on erythrocyte membranes was not affected by BBG, both, PPADS and MRS2159 had a significant effect, providing an explanation for the reduction of Hla-dependent hemolysis by these compounds (Figure 5).

### 2.4. P2X-Receptor-Antagonists Inhibit Hla-Dependent Calcein Release from Liposomes and Formation of Oligomers

**Figure 6:**
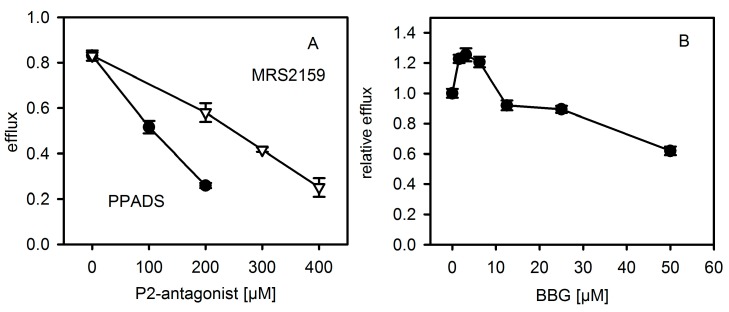
P2XR-antagonists reduce Hla-dependent efflux of calcein from liposomes. Efflux of calcein from liposomes was measured after 30 min at 37 °C. The concentration of Hla is about 250 nM; antagonist concentration is as indicated. Panel (**A**): PPADS (pyridoxal phosphate-6-azo benzene-2,4-disulfonic acid) and PPADS derived inhibitor MRS2159. Panel (**B**): BBG (Brilliant Blue G); here the efflux in presence of BBG is normalized to the value in absence of BBG. Data are triplicates each.

**Figure 7:**
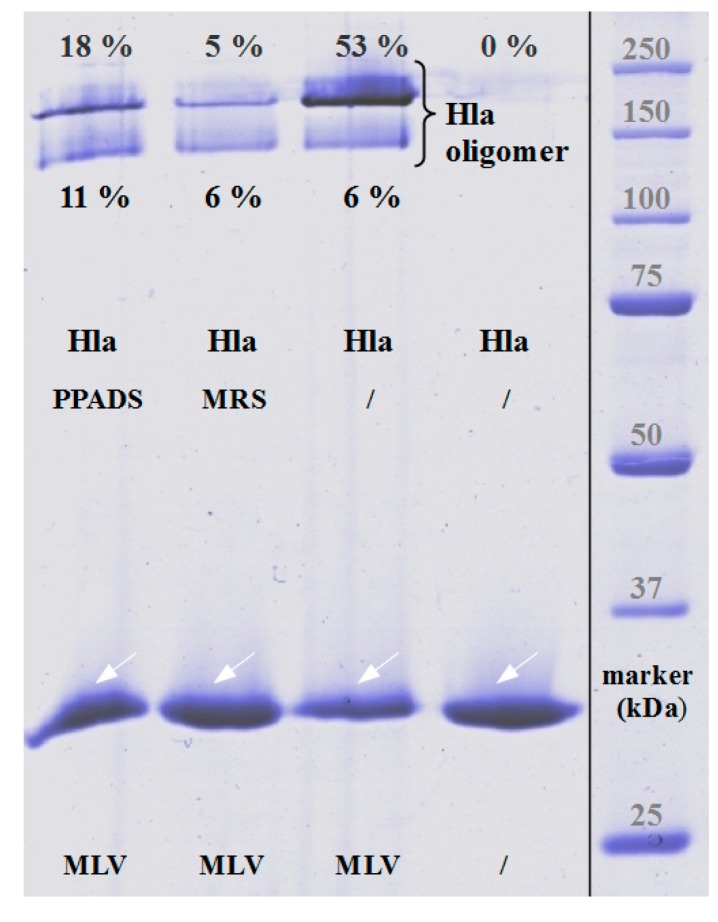
Direct influence of P2XR-inhibitors on Hla-oligomer formation on liposomes. SDS-gel electrophoresis was performed to estimate the amount of oligomerised Hla, which runs as high-molecular weight bands in absence of sample heating and reduced SDS-concentration. The white arrows indicate the position of Hla-monomers, the black bracket SDS-stable Hla-oligomers. Overall oligomer formation on multilamellar liposomes (MLVs) is reduced from 59% (without inhibitor treatment) to 29% in case of PPADS and 11% for MRS, respectively. The Hla concentration is 2.5 µM in each sample.

In order to exclude that antagonists might directly affect the interaction of Hla with a pure lipid membrane, we studied calcein efflux from unilamellar liposomes. Liposomes containing 40% cholesterol, 20% eSM (egg yolk N-acyl-d-sphingosine-1-phosphocholine), 20% ePE (egg yolk 1,2-diacyl-*sn*-glycero-3-phosphoethanolamine) and 20% bPS (1,2 diacyl-*sn*-glycero-3-phospho-l-serine from bovine brain) were generated and filled with self-quenching concentrations of calcein. Hla-dependent efflux of the dye was detected by means of increasing fluorescence due to de-quenching upon release from the liposomes. Strikingly, PPADS, MRS2159 and BBG all inhibited Hla-dependent calcein efflux (Figure 6). In the case of PPADS, the concentration required was similar to the one found for hemolysis of rabbit erythrocytes, differing not more than a factor of two; in the case of MRS2159, the required dose was slightly higher, whereas, for BBG, the effective concentration was about 5-fold of the inhibitory dose. Importantly, none of the three antagonists caused calcein efflux in the absence of Hla (data not shown). Gel-electrophoresis of multilamellar liposomes (MLVs) incubated with Hla in the absence or presence of PPADS or MRS2159 revealed that reduced lysis nicely correlates with a reduction of oligomer-formation (Figure 7).

### 2.5. Evidence for Direct Hla-PPADS Interaction

**Figure 8:**
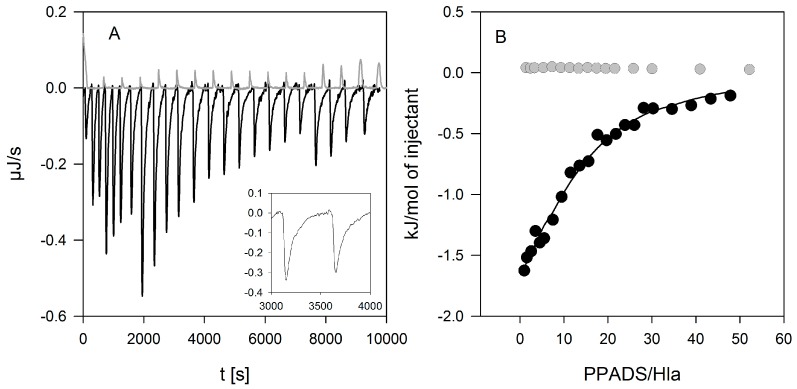
Binding of PPADS to Hla detected by isothermal titration calorimetry. PPADS (5 mM) was injected into Hla (18 µM) resulting in exothermic reaction enthalpy (black lines (**A**) and symbols (**B**)), clearly differing from the slightly positive heat of dilution of PPADS (gray lines (**A**) and symbols (**B**)). The peaks were rather broad (inset in panel (**A**)), indicating a slow response to PPADS binding. A fit based on one binding site per Hla (solid line in panel (**B**)) yielded a binding constant of 2200 ± 100 M^−1^ and a binding enthalpy of 190 ± 1 kJ/mol.

Next, we investigated whether PPADS can directly interact with Hla. Indeed, binding of PPADS to Hla could be readily detected by isothermal titration calorimetry (ITC, Figure 8). The affinity determined in four experiments at 20 °C was 3700 ± 1200 M^−1^, with a binding enthalpy of −150 ± 30 kJ/mol. Two curves measured at 37 °C yielded similar binding constants (3200 and 4200 M^−1^) and a somewhat lower binding enthalpy (−39 and −46 kJ/mol). For the analysis, a stoichiometry of *n* = 1 was assumed since the low affinity does not allow to determine this value independently.

**Figure 9:**
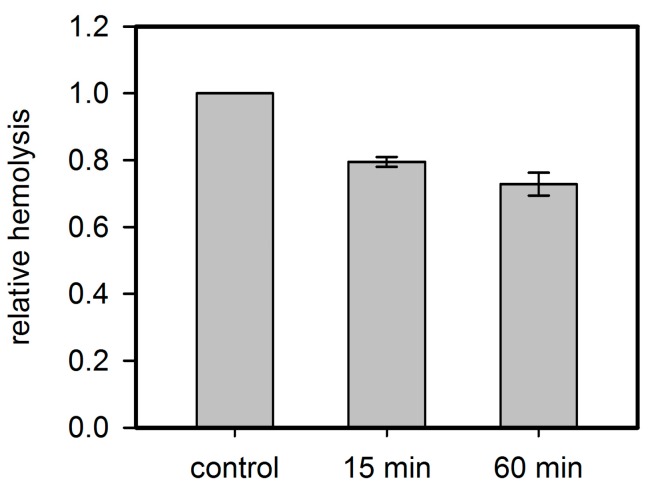
Residual inhibition of rabbit erythrocyte hemolysis by Hla pre-treated with PPADS. Hla (2.5 µM) and PPADS (1 mM) were incubated for 15 and 60 min on ice. Then, the solutions were diluted 1000-fold for use in the standard hemolysis assay. Hemolysis level is shown relative to the control, which received the same treatment with buffer instead of PPADS. Despite the low concentration of PPADS in the assay, a considerable inhibition is observed, indicating that PPADS indeed interacts with Hla.

In order to prove that Hla-binding to PPADS contributes to the inhibitory effect of the antagonist; toxin was incubated with PPADS at high concentrations (2.5 µM Hla and 1 mM PPADS) for 15 and 60 min, and diluted for use in the standard hemolysis assay. The final concentration of PPADS in the assay was about 1 µM, a level at which the substance is not inhibitory any more (Figure 2). A clear inhibition was observed despite the low PPADS concentration, and the effect increases upon increasing incubation time (Figure 9). Thus, binding of PPADS reduces the active fraction of Hla, possibly in an irreversible manner.

## 3. Discussion

Previous observations that P2XR-antagonists inhibit hemolysis caused by several pore forming proteins, including Hla [5,6,8,9,13,14], led to the now widely accepted idea that P2XRs are critical for pore-forming toxin-dependent hemolysis [4]. This role has been discussed as one of the main functions of these receptors in erythrocytes [15]. In this context, it is upheld that a few small toxin pores are insufficient to directly cause hemolysis, whereas the release of ATP, and the subsequent activation of P2XRs would trigger substantial ion fluxes, colloid-osmotic swelling of cells, and would ultimately lead to hemolysis.

This hypothesis is based on the reduction of hemolysis in the presence of ATP-converting enzymes or P2XR-antagonists, and knock-down experiments [5,6,8,9]. Interestingly, data reporting an increase of toxin-induced hemolysis due to presence of ATP can only be found for Hla and LTX [6,8] and not for other agonists of P2XR. In absence of toxins activation of P2XR-receptors by the addition of ATP to the extracellular medium leads to flux of monovalent ions and to PS-exposure, but not to significant levels of hemolysis at the time scale of typical hemolysis experiments, namely up to 60 min [16]. Thus, P2XR-receptor activation alone does not lead to hemolysis, indicating that the toxin pores themselves must fulfill a significant part in destabilizing membrane stability and ion homeostasis. In case of Hla, the increase in hemolysis due to presence of 3 mM ATP occurred exclusively at very early points in time (2.5 min): here, the hemolysis level increased from 4% to 6%. Later on, a decrease in hemolysis was observed [8]. In contrast, an about 3-fold increase of hemolysis at 1 mM ATP after 60 min incubation was reported for human erythrocytes exposed to LTX. To sum up, a general effect of extracellular ATP on PFT-dependent hemolysis has not been unequivocally documented.

The fact that our data with rabbit erythrocytes deviate in some aspects from an earlier study might in part be related to the different purity levels of the Hla-preparations used: whereas, our preparations from cultures of *S. aureus* contain very low amounts of contaminants (see Figure 5, last lane shows Hla without erythrocytes), Hla from a commercial source consists of merely ~60% protein (according to the manufacturer). Moreover, only part of said protein content appears to be intact Hla [17]; in the same study, it was shown that even heat-inactivated *S. aureus* preparations—thus, being devoid of functional Hla-amplify the response to Hla-treatment in monocytes. Some variation in modulation of hemolytic activity has also been observed for HlyA, when crude and pure preparations are compared [5].

The results presented here cast doubt on the proposed role of P2XR-activation in enhancing hemolysis of erythrocytes by Hla. Previous results thought to support this concept could actually be explained by non-canonical effects of inhibitors. First, PPADS and MRS2159 do not only block Hla-dependent hemolysis, but markedly reduce calcein release from liposomes. Second, the oligomerisation of Hla is drastically reduced by PPADS, as shown on nucleated cells, erythrocytes, and liposomes. Finally, a direct interaction between Hla and PPADS could be revealed by ITC. Notably, reduction of the inhibitory action on Hla persisted after dilution of PPADS following treatment.

Our collective results suggest an interference of PPADS and MRS2159 with Hla binding and oligomer formation, apparently due to the competition between PPADS and membrane constituents for Hla. This nicely fits to the observation that binding of Hla to human erythrocytes—which have an overall lower affinity for Hla—is much more affected by PPADS than binding to rabbit erythrocytes, which carry binding sites of higher affinity [10]. Inhibition by BBG seems to operate by a different mechanism. The minor effect observed due to the presence of oxATP is not necessarily related to P2XR-inactivation; as it has been shown to be not a specific inhibitor of P2XRs [18,19] and the effects might be due to the inhibition of other targets.

To our knowledge, the impact of P2-receptor antagonists on pore formation on pure lipid membranes has only been tested thus far on black lipid membranes; and merely RTX-toxins were studied [13]. In that work, membrane perforation appeared to not be altered. Yet, the applied concentrations of PPADS might have been simply too low, since under these conditions hemolysis was inhibited by merely 50%. Also, the mode of pore formation by RTX-toxins is likely to be quite different from pore formation by Hla. It is well possible that antagonists do not interact with RTX, but with structurally unrelated Hla. A number of small molecular weight compounds have been identified that seem to interfere with the oligomerisation of Hla [20,21,22], but we are not aware of compounds interfering with RTX-toxin activity.

The present work sheds light on how P2XR-antagonists interfere with the function of a membrane pore-forming toxin through non-canonical mechanisms. The data warrant a critical reassessment of the widely accepted idea that P2XRs are general enhancers of membrane pore formation, and thus they call for further studies in this field.

## 4. Materials and Methods

### 4.1. Chemicals

Egg yolk 1,2-diacyl-sn-glycero-3-phosphoethanolamine (ePE), 1,2 -diacyl-sn-glycero-3-phospho-L-serine from bovine brain (bPS), egg yolk N-acyl-d-sphingosine-1-phosphocholine (eSM), and cholesterol (Chol) were from Sigma Aldrich (Deisenhofen, Germany). N-(9Z-octadecenoyl)-sphing-4-enine-1-phosphocholine (OSM) was purchased from Avanti Polar Lipids (Alabaster, AL, USA). All of the lipids were already dissolved in chloroform except for cholesterol, which was obtained as powder and dissolved in chloroform for use. All of the chemicals for gel-electrophoresis and buffer solutions were purchased from Roth GmbH (Karlsruhe, Germany). Hexokinase, PPADS (pyridoxal phosphate-6-azophenyl-2′,4′-disulfonic acid, tetrasodium salt hydrate), calcein, oxATP, BBG (Brilliant Blue G), and MRS2159 (a derivative of PPADS) were obtained from Sigma Aldrich (Deisenhofen, Germany). ATP Bioluminescence Assay Kit CLSII was purchased from ROCHE (Mannheim, Germany).

### 4.2. Toxin (Hla)

Except for fluorescence microscopy all of the experiments were carried out with wild type Hla, which was purified from supernatant of *S. aureus* cultures (Hla-deficient strain DU1090 transformed with Hla-encoding plasmid pAC), as previously described [23]. For fluorescence microscopy, the mutant S3C was labeled with 5-iodoacetamidofluorescein (5-IAF, Molecular Probes, Eugene, OR, USA) according to the protocol described earlier [23]. Internally labeled toxin (^35^S-Hla) was prepared as described before [24].

### 4.3. ATP-Assay

Intracellular ATP levels in cells were measured as described elsewhere [11]. In brief, HaCaT-cells were seeded at a density of 10^4^ cells per well in 96-well tissue culture plates. The next day, treatments were performed and ATP was measured using the ATP Bioluminescence Assay Kit CLSII from ROCHE. The samples were analyzed with a Lumat 9705 instrument from Berthold (Bad Wildbad, Germany).

### 4.4. Oligomerisation of Hla on HaCaT-Cells

The basic procedure was as described elsewhere [3]. To assess the impact of PPADS on binding and oligomerisation of Hla on nucleated cells, we used HaCaT-cells, human non-virally transformed keratinocytes [25]. The cell line, obtained from Deutsche Sammlung von Mikroorganismen und Zellkulturen (DSMZ, Braunschweig, Germany), was free of mycoplasma. Cells were plated on tissue culture dishes 24 h before start of the experiment. After the addition of 1 mM PPADS, plates were incubated at 37 °C for 30 min. Then, the plates were placed on ice and ^35^S-Hla (~30 nM) was added. After 40 min incubation on ice, cells were briefly washed. Directly after washing (0 min) or after 15 min of incubation at 37 °C, bound toxin was determined. Cell surface protein labeling (CSPL) with biotin and subsequent precipitation via Neutravidin-pull-down was employed to detect Hla on the cell surface, while immunoprecipitation (IP) with Hla-antibody was used to detect total Hla, i.e., toxin associated with both cell surface and cytosol. Neutravidin-precipitates were separated by SDS-PAGE and ^35^S-Hla-containing bands, representing monomers and SDS-stable oligomers, were visualized by fluorography. Samples for SDS-PAGE were heated to 60 °C for 10 min prior to loading.

### 4.5. Erythrocytes and Hemolysis Assay

Rabbit erythrocytes were purchased from Fiebig Nährstofftechnik (Idstein, Germany). Human erythrocytes were collected from leucocyte-enriched buffy coats of healthy donors (Transfusion Center Mainz, Germany) and were kindly provided by Dr. Iris Bellinghausen (Department of Dermatology, University Medical Center Mainz, Germany). Informed consent was obtained from all of the donors. All of the experiments with erythrocytes were performed in a buffer containing 14 mM Hepes, 0.8 mM MgSO_2_, 1.8 mM CaCl_2_, 5.3 mM KCl, 124 mM NaCl, 5.6 mM glucose, titrated with NaOH to obtain a pH of 7.2 at 37 °C. Erythrocytes were washed by centrifugation (4 °C, 500 g) in this buffer until the supernatant was clear. For the hemolysis assay, the erythrocytes were diluted to about 2.5% yielding an OD of ~0.8 at 415 nm and 1 mm path-length for fully lysed samples. The components of the assay were mixed directly before starting the experiment. The erythrocytes were added at the end at a sufficiently large volume to avoid local concentration effects. Samples were shaken at 200 rpm for 60 min at 37 °C, transferred to ice, centrifuged (4 °C, 500 g) and extracellular hemoglobin in the supernatant was quantified by absorption spectroscopy (NanoDrop ND-1000 Spectrophotometer, peqLab, VWR International, LLC). Hemolysis was determined based on the concentration of hemoglobin measured at 415 nm. At this wavelength, absorption of BBG at the concentrations used is negligible. In the case of PPADS and MRS2159 there is a measurable contribution at this wavelength, but in the concentrations used the contribution it is not very high and could be subtracted by preparing appropriate controls. Additionally, the results were checked by comparison with determination of hemoglobin concentration at 576 nm. Erythrocytes incubated in absence of Hla served as negative control (0% hemolysis), while erythrocytes osmotically lysed by dilution with water served as positive control (100% hemolysis) at the corresponding wavelength. Due to the lack of effect upon the addition of hexokinase, the activity of hexokinase was checked and found to be about 60% of the level reported by the supplier. The buffer for the experiments in the presence of hexokinase contained increased levels of glucose (10 mM) and MgSO_4_ (2.5 mM). In order to maintain osmolarity, the concentration of NaCl was adjusted accordingly. For the experiments addressing the residual activity of PPADS after dilution to non-active concentrations, PPADS (1 mM) and Hla (2.5 µM) were incubated on ice for the time given. Then, the solution was diluted quickly 1000-fold in two steps, and the diluted solution was incubated with erythrocytes and was prepared for determination of free hemoglobin, as described above. Hla (2.5 µM) incubated in buffer and treated exactly the same way as the PPADS-containing sample served as reference.

### 4.6. Hla-Oligomerisation on Pure Lipid Membranes (MLVs) and Erythrocytes

MLV preparation was adapted from the thin-film method [26]. A “pseudo ternary” lipid mixture [27] made of 40% cholesterol, 20% OSM, 20% ePE, and 20% bPS with a total lipid amount of 1mg was prepared in chloroform. After chloroform evaporation under nitrogen, the lipid film was dried for 3 h under vacuum. The lipid film was then rehydrated by addition of 1 mL 70 mM sodium-phosphate-buffer (pH 7.2). 20 µL of this MLV-solution were mixed with 20 µL solution containing 2.5 µM Hla in absence or presence of 500 µM MRS or PPADS, respectively. After 30 min of incubation at room temperature, the samples were subjected to SDS-gel-electrophoresis [28] with some modification: the samples were not heated prior to loading, the loading buffer does not contain SDS, and the running buffer contained only 0.1% SDS. Under these conditions, monomeric Hla runs true in the gel, but Hla-heptamers (pores and presumably also some types of pre-pores) retain their oligomeric structure and lead to characteristic high molecular bands. Finally, the gels were stained according to [29].

Oligomer formation on erythrocytes was determined similarly, employing 0.5% human or rabbit erythrocytes in phosphate buffered saline, which were incubated with 2.5 µM Hla in a final volume of 40 µL for 30 min at room temperature in the presence or absence of P2XR-inhibitors, as indicated. Erythrocytes were then concentrated by centrifugation with 1000 g for 15 min at 4 °C. The supernatant was discarded and the erythrocyte-pellet was supplemented with SDS loading puffer containing 2% SDS in final. The samples were loaded on 10% SDS-gels for electrophoresis, without prior heat-denaturation.

### 4.7. Hla-Induced Calcein Efflux

Liposomes (LUVs, 100 nm diameter) were prepared, as described before [27], using a solution of 50 mM calcein and 70 mM phosphate-buffer, pH 7.2 (final) as hydration buffer. Free calcein was removed with a desalting column (PD10, Amersham Biosciences Europe GmbH, Freiburg, Germany). Efflux experiments were performed with a lipid concentration of about 50 µM (20 mol % eSM, 40 mol % cholesterol, 20 mol % ePE, 20 mol % bPS) and 200–300 nM Hla, with P2XR-inhibitor concentrations, as indicated. Liposomes were added at the end into the assay mixture. Fluorescence change due to the dequenching of calcein was followed at 515 nm (excitation 495 nm) with a Hitachi F4500 (Binninger Analytic, Schwaebisch Gmuend, Germany) at 37 °C for 30 min. The level of fluorescence at full leakage was determined by the addition of Triton-X at a final concentration of 2%. Dilution and the inner-filter effect due to the absorbance of P2XR-antagonists was taken into account.

### 4.8. Fluorescence Microscopy

Fluorescence micrographs were taken with a Keyence BZ 8000K (Keyence Corporation, Osaka, Japan). Samples were incubated in reaction tubes at room temperature before observation. The high amount of Hla (2.5 µM) was necessary to yield a detectable fluorescence signal. Under these conditions, both types of erythrocytes were already lysed when investigated. After the indicated times, an aliquot of the sample was transferred to the slide, covered and observed after the erythrocytes had settled, which takes about 5 min. From the micrographs, which contained many erythrocytes, four individual, representative cells were selected for presentation. In case of rabbit erythrocytes, the population was rather heterogeneous, thus cells with different levels of fluorescence were included.

### 4.9. Isothermal Titration Calorimetry

Hla at a concentration between 10 and 30 µM in the cell was titrated with PPADS (2.5–5 mM) in a 250 µL syringe, both dissolved in 70 mM sodium-phosphate buffer (VP-ITC, Malvern, Worcestershire, UK) at 20 and 37 °C. PPADS was injected in 3–10 μL-steps, at a stirring speed of 200 rpm, with a reference power of 60 μJ/s, and a filter period of 2 s. Baselines were set automatically by the software, and re-adjusted manually. Peak integration and calculation of [PPADS]/[Hla] was performed automatically by the software. Corresponding baselines (titration of PPADS in buffer) were subtracted; titration of buffer in protein did not yield measureable heats.

Single experiments were analyzed based on a model with one binding site for PPADS on a Hla monomer. The error for the parameters of a single experiment is given as evaluated by the fitting routine included in the instrument (OriginLab). Finally, the results of three experiments were averaged and the SEM is given.

### 4.10. Statistics

For the hemolysis experiments, between two and five curves were averaged. The error bars correspond to the standard error of the mean; in the case of two replicates it is the deviation of the values from the mean. For each individual curve, the extracellular hemoglobin concentration was measured twice. Control experiments (absence of inhibitors) were measured the same day, mostly directly in parallel with the inhibited sample. The effect of the three inhibitors was consistently observed in experiments employing other erythrocytes charges. Liposomal efflux experiments are the average of three experiments with the same liposome preparation.

## References

[1] Iacovache I., Van der Goot F.G., Pernot L. (2008). Pore formation: An ancient yet complex form of attack. Biochim. Biophys. Acta.

[2] Dal Peraro M., Van der Goot F.G. (2016). Pore-forming toxins: Ancient, but never really out of fashion. Nat. Rev. Microbiol..

[3] Husmann M., Beckmann E., Boller K., Kloft N., Tenzer S., Bobkiewicz W., Neukirch C., Bayley H., Bhakdi S. (2009). Elimination of a bacterial pore-forming toxin by sequential endocytosis and exocytosis. FEBS Lett..

[4] Skals M., Praetorius H.A. (2013). Mechanisms of cytolysin-induced cell damage—A role for auto- and paracrine signalling. Acta Physiol..

[5] Skals M., Jorgensen N.R., Leipziger J., Praetorius H.A. (2009). Alpha-hemolysin from Escherichia coli uses endogenous amplification through P2X receptor activation to induce hemolysis. Proc. Natl. Acad. Sci. USA.

[6] Munksgaard P.S., Vorup-Jensen T., Reinholdt J., Soderstrom C.M., Poulsen K., Leipziger J., Praetorius H.A., Skals M. (2012). Leukotoxin from Aggregatibacter actinomycetemcomitans causes shrinkage and P2X receptor-dependent lysis of human erythrocytes. Cell. Microbiol..

[7] Skals M., Bjaelde R.G., Reinholdt J., Poulsen K., Vad B.S., Otzen D.E., Leipziger J., Praetorius H.A. (2014). Bacterial RTX toxins allow acute ATP release from human erythrocytes directly through the toxin pore. J. Biol. Chem..

[8] Skals M., Leipziger J., Praetorius H.A. (2011). Haemolysis induced by alpha-toxin from Staphylococcus aureus requires P2X receptor activation. Pflug. Arch-Eur. J. Physiol..

[9] Hejl J.L., Skals M., Leipziger J., Praetorius H.A. (2013). P2X receptor stimulation amplifies complement-induced haemolysis. Pflug. Arch-Eur. J. Physiol..

[10] Hildebrand A., Pohl M., Bhakdi S. (1991). Staphylococcus aureus alpha-toxin. Dual mechanism of binding to target cells. J. Biol. Chem..

[11] Haugwitz U., Bobkiewicz W., Han S.R., Beckmann E., Veerachato G., Shaid S., Biehl S., Dersch K., Bhakdi S., Husmann M. (2006). Pore-forming Staphylococcus aureus alpha-toxin triggers epidermal growth factor receptor-dependent proliferation. Cell. Microbiol..

[12] Nagahama M., Seike S., Shirai H., Takagishi T., Kobayashi K., Takehara M., Sakurai J. (2015). Role of P2X7 receptor in Clostridium perfringens beta-toxin-mediated cellular injury. Biochim. Biophys. Acta.

[13] Masin J., Fiser R., Linhartova I., Osicka R., Bumba L., Hewlett E.L., Benz R., Sebo P. (2013). Differences in purinergic amplification of osmotic cell lysis by the pore-forming RTX toxins Bordetella pertussis CyaA and Actinobacillus pleuropneumoniae ApxIA: The role of pore size. Infect. Immun..

[14] Fagerberg S.K., Skals M., Leipziger J., Praetorius H.A. (2013). P2X receptor-dependent erythrocyte damage by alpha-hemolysin from Escherichia coli triggers phagocytosis by THP-1 cells. Toxins.

[15] Sluyter R. (2015). P2X and P2Y receptor signaling in red blood cells. Front. Mol. Biosci..

[16] Sluyter R., Shemon A.N., Hughes W.E., Stevenson R.O., Georgiou J.G., Eslick G.D., Taylor R.M., Wiley J.S. (2007). Canine erythrocytes express the P2X7 receptor: Greatly increased function compared with human erythrocytes. Am. J. Physiol..

[17] Craven R.R., Gao X., Allen I.C., Gris D., Bubeck Wardenburg J., McElvania-Tekippe E., Ting J.P., Duncan J.A. (2009). Staphylococcus aureus alpha-hemolysin activates the NLRP3-inflammasome in human and mouse monocytic cells. PloS ONE.

[18] Anderson C.M., Nedergaard M. (2006). Emerging challenges of assigning P2X7 receptor function and immunoreactivity in neurons. Trends Neurosci..

[19] Beigi R.D., Kertesy S.B., Aquilina G., Dubyak G.R. (2003). Oxidized ATP (oATP) attenuates proinflammatory signaling via P2 receptor-independent mechanisms. Br. J. Pharmacol..

[20] Qiu J., Niu X., Dong J., Wang D., Wang J., Li H., Luo M., Li S., Feng H., Deng X. (2012). Baicalin protects mice from Staphylococcus aureus pneumonia via inhibition of the cytolytic activity of α-hemolysin. J. Infect. Dis..

[21] Dong J., Qiu J., Zhang Y., Lu C., Dai X., Wang J., Li H., Wang X., Tan W., Luo M. (2013). Oroxylin A inhibits hemolysis via hindering the self-assembly of alpha-hemolysin heptameric transmembrane pore. PLoS Comput. Biol..

[22] Wang J., Zhou X., Liu S., Li G., Shi L., Dong J., Li W., Deng X., Niu X. (2015). Morin hydrate attenuates Staphylococcus aureus virulence by inhibiting the self-assembly of alpha-hemolysin. J. Appl. Microbiol..

[23] Palmer M., Jursch R., Weller U., Valeva A., Hilgert K., Kehoe M., Bhakdi S. (1993). Staphylococcus aureus alpha-toxin. Production of functionally intact, site-specifically modifiable protein by introduction of cysteine at positions 69, 130, and 186. J. Biol. Chem..

[24] Walker B., Krishnasastry M., Zorn L., Bayley H. (1992). Assembly of the oligomeric membrane pore formed by Staphylococcal alpha-hemolysin examined by truncation mutagenesis. J. Biol. Chem..

[25] Boukamp P., Petrussevska R.T., Breitkreutz D., Hornung J., Markham A., Fusenig N.E. (1988). Normal Keratinization in a spontaneously immortalized Aneuploid Human Keratinocyte cell-line. J. Cell Biol..

[26] Bangham A.D., Standish M.M., Watkins J.C. (1965). Diffusion of univalent ions across the lamellae of swollen phospholipids. J. Mol. Biol..

[27] Schwiering M., Brack A., Stork R., Hellmann N. (2013). Lipid and phase specificity of alpha-toxin from *S. aureus*. Biochim. Biophys. Acta.

[28] Laemmli U.K. (1970). Cleavage of structural proteins during the assembly of the head of bacteriophage T4. Nature.

[29] Kang D.H., Gho Y.S., Suh M.K., Kang C.H. (2002). Highly sensitive and fast protein detection with coomassie brilliant blue in sodium dodecyl sulfate-polyacrylamide gel electrophoresis. Bull. Korean Chem. Soc..

